# Study on the Efficiency and Mechanism of a Novel Copper-Based Composite Material Activated by Supramolecular Self-Assembly for Degrading Reactive Red 3BS

**DOI:** 10.3390/nano16020111

**Published:** 2026-01-15

**Authors:** Jiangming Dai, Xinrong Wang, Bo Chen, Liang Chen

**Affiliations:** 1 School of Biology and Environment, Zhejiang Wanli University, Ningbo 315100, China; 2023881109@zwu.edu.cn (J.D.); 2024881013@zwu.edu.cn (X.W.); 2 Key Laboratory of Public Health Security, Ministry of Education, School of Public Health, Fudan University, Shanghai 200032, China; chenb@fudan.edu.cn

**Keywords:** supramolecular self-assembly, novel copper-based composite, sodium bicarbonate-activated hydrogen peroxide, degradation of Reactive Red 3BS

## Abstract

To address the challenge of treating refractory organic dyes in textile wastewater, this study synthesized a novel copper-based composite material (designated MEL-Cu-6HNA) via a supramolecular self-assembly–pyrolysis pathway. Its core component consists of CuO/Cu_2_O(SO_4_), which was applied to efficiently degrade the Reactive Red 3BS dye within a sodium bicarbonate-activated hydrogen peroxide (BAP) system. This material was applied to degrade the Reactive Red 3BS dye using a sodium bicarbonate-activated hydrogen peroxide system. The morphology, crystal structure, and surface chemistry of the material were systematically characterized using scanning electron microscopy (SEM), X-ray diffraction (XRD), Fourier transform infrared spectroscopy (FTIR), and X-ray photoelectron spectroscopy (XPS). Electron paramagnetic resonance (EPR) was employed to identify reactive species generated during the reaction. The effects of dye concentration, H_2_O_2_ concentration, MEL-Cu-6HNA dosage, and coexisting substances in water on degradation efficiency were systematically investigated, with active species identified via EPR. This study marks the first application of the supramolecular self-assembled CuO/Cu_2_O(SO_4_)_2_ composite material MEL-Cu-6HNA, prepared via pyrolysis, in a sodium bicarbonate-activated hydrogen peroxide system. It achieved rapid and efficient decolorization of the recalcitrant Reactive Red 3BS dye. The three-dimensional sulfate framework and dual Cu^2+^ sites of the material significantly enhanced the degradation efficiency. MEL-Cu-6HNA achieved rapid and efficient decolorization of the recalcitrant Reactive Red 3BS in a sodium bicarbonate-activated hydrogen peroxide system. The material’s three-dimensional sulfate framework and dual Cu^2+^ sites significantly enhanced interfacial electron transfer and Cu^2+^/Cu^+^ cycling activation capacity. ·OH served as the primary reactive oxygen species (ROS), with SO_4_^2−^, ^1^O_2_, and ·O_2_^−^ contributing to sustained radical generation. This system achieved 95% decolorization within 30 min, demonstrating outstanding green treatment potential and providing a reliable theoretical basis and practical pathway for efficient, low-energy treatment of dyeing wastewater.

## 1. Introduction

With the rapid development of the chemical, textile, and dyeing industries, reactive dyes have been widely applied across multiple sectors including textiles, food, plastics, printing, leather, cosmetics, and pharmaceuticals due to their strong coloring power and excellent lightfastness [1,2,3]. Among these, Reactive Red 3BS is a typical azo-based reactive dye. Its molecular structure contains numerous aromatic rings and azo groups, endowing it with exceptional chemical stability and resistance to degradation in aqueous environments [4]. Direct discharge of wastewater containing substantial amounts of Reactive Red 3BS into natural water bodies causes turbidity and inhibits photosynthesis. Moreover, its degradation products often contain carcinogenic or mutagenic aromatic amine compounds, posing significant ecological and health risks [5]. Therefore, there is an urgent need to develop efficient, green, and cost-effective technological approaches to achieve complete mineralization of Reactive Red 3BS, meeting the pressing demands for sustainable water environment management.

Among various advanced oxidation processes (AOPs), the bicarbonate-activated peroxide (BAP) system has garnered widespread attention due to its readily available raw materials, simple operation, and environmental friendliness [6]. The core mechanism involves the synergistic generation of potent oxidative radicals from NaHCO_3_ and H_2_O_2_, primarily hydroxyl radicals (·OH), percarbonate radicals (HCO_3_·), and superoxide anions (O_2_·^−^) [7]. These reactive species efficiently degrade recalcitrant organic pollutants like dyes, aromatic amines, and phenols under ambient conditions, achieving near-complete mineralization (CO_2_, H_2_O, and inorganic acids). The performance of BAP systems can be significantly enhanced through synergistic catalysis by transition metal ions or metal oxides [8]. Studies indicate that trace amounts of metal ions such as Cu^2+^, Co^2+^, and Fe^3+^ accelerate H_2_O_2_ decomposition, generating increased ·OH radicals while promoting electron transfer between HCO_3_^−^ and H_2_O_2_. This process forms highly reactive percarbonate radicals and superoxide radicals [9,10,11]. In practical dye wastewater treatment, adding an appropriate amount of metal catalyst enables the BAP system to achieve over 90% color removal and significant Chemical oxygen demand (COD)/Total organic carbon (TOC) reduction within 30 min–1 h, while maintaining good catalyst lifetime under neutral or weakly alkaline pH conditions.

Among metal oxide catalysts, copper-based materials offer distinct advantages. Copper is abundant in nature and cost-effective. Its ability to convert between Cu^+^ and Cu^2+^ states enables it to serve as both an electron donor (Cu^+^) and electron acceptor (Cu^2+^) during H_2_O_2_ decomposition, forming a closed-loop electron transfer pathway that continuously generates ·OH and ·O_2_^−^ radicals. Literature reports indicate that monophasic CuO significantly enhances H_2_O_2_ decomposition rates in BAP systems, while Cu_2_O (or its sulfate-derived phase) exhibits higher activity in capturing ·O_2_^−^ and ·CO_3_^−^ due to its lower oxidation state (Cu^+^) [12]. Therefore, constructing a composite phase where Cu^+^ and Cu^2+^ coexist is expected to achieve synergistic ROS generation without introducing additional additives, thereby enhancing the degradation efficiency of Reactive Red 3BS. The key to achieving this goal lies in the controllability of the material structure. Traditional high-temperature solid-state methods often result in coarse particles, low specific surface area, and difficulty in achieving precise Cu^+^/Cu^2+^ ratios [13]. Supramolecular self-assembly, as a bottom-up organization strategy, utilizes noncovalent interactions such as hydrogen bonds, coordination bonds, and π-π stacking to pre-arrange small molecules or ionic units into ordered precursor networks in solution [14,15]. Subsequent thermal treatment converts these ordered precursors into metal oxides, enabling composite-scale grain growth while preserving the original spatial arrangement. This significantly enhances specific surface area and phase boundary density. 6-hydroxynicotinic acid (6-HNA) was chosen as the coligand because its pyridine-nitrogen, carboxyl, and hydroxyl groups provide polydentate coordination sites and abundant hydrogen bond donors and acceptors. This is essential for the construction of a stable 3D supramolecular framework with melamine. In recent years, supramolecular self-assembly has been successfully applied to prepare copper-based composite clusters, copper coordination polymers, and multiphase copper oxide composites, demonstrating its unique advantages in regulating metal valence states and enhancing optoelectronic/catalytic activity [16].

This study proposes a copper-based composite material based on supramolecular self-assembly. Through a supramolecular self-assembly-pyrolysis pathway, a CuO/Cu_2_O (SO_4_) composite material was produced, with coexisting Cu^+^/Cu^2+^ states, through a supramolecular self-assembly-pyrolysis pathway. We systematically elucidated its efficient degradation behavior of Reactive Red 3BS and radical generation mechanism in a NaHCO_3_^−^-activated H_2_O_2_ (BAP) system, providing new material insights and experimental evidence for achieving green and efficient dye wastewater treatment.

## 2. Materials and Methods

### 2.1. Preparation of MEL-Cu-6HNA Material

**Figure 1:**
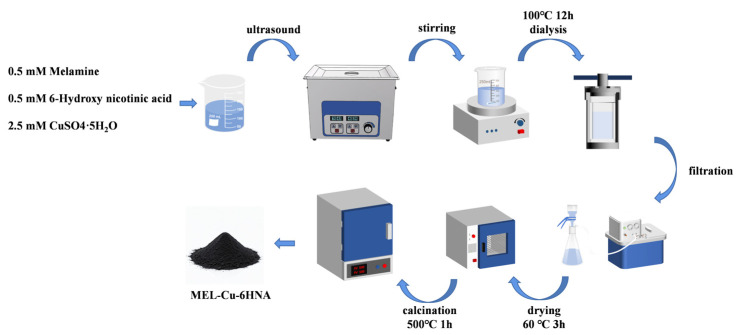
Preparation diagram of MEL-Cu-6HNA composite material.

**Scheme 1:**
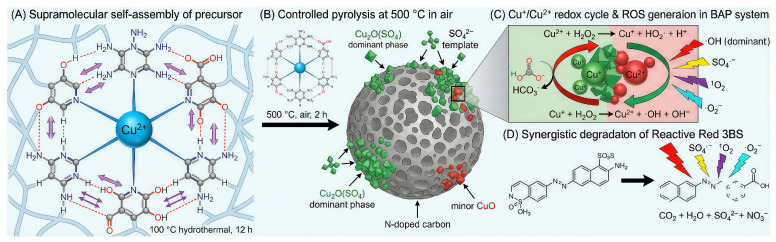
Proposed supramolecular self-assembly mechanism and pyrolysis transformation pathway of the MEL-Cu-6HNA precursor; (**A**) Molecular-level interactions; (**B**) Formation of the 3D supramolecular precursor framework; (**C**) Pyrolysis transformation to the final composite material; (**D**) Synergistic degradation of Reactive Red 3BS.

Dissolve 0.5 mmol melamine, 0.5 mmol 6-hydroxynicotinic acid, and 2.5 mmol copper sulfate pentahydrate in a mixed solvent of N,N-dimethylformamide (DMF) and deionized water (volume ratio 2:1). After ultrasonic dispersion for 10 min, mix the solution with magnetic stirring for 30 min. The mixture was transferred into a PTFE-lined stainless steel autoclave and subjected to hydrothermal reaction at 100 °C for 12 h. After natural cooling to room temperature, the precipitate was collected. The precipitate was washed three times with deionized water and dried in a 60 °C oven for 3 h to obtain the precursor product. The precursor was placed in a muffle furnace and heated to 500 °C in air at a rate of 5 °C/min, held at this temperature for 1 h, yielding the final composite material sample designated MEL-Cu-6HNA. As shown in Figure 1, the complete preparation process is clearly illustrated. The proposed formation mechanism (illustrated in Scheme 1) involves cooperative coordination and non-covalent interactions. Melamine and 6-hydroxynicotinic acid self-assemble with Cu^2+^ through multiple coordination sites, reinforced by extensive hydrogen bonding and π–π stacking, yielding a highly ordered supramolecular precursor. During subsequent pyrolysis, sulfate ions serve as structural templates that prevent complete oxidation of copper, favoring the formation of a Cu^+^-rich Cu_2_O(SO_4_) phase alongside minor CuO.

### 2.2. Characterization of MEL-Cu-6HNA

The morphology of the MEL-Cu-6HNA composite was investigated using scanning electron microscopy (SEM) imaging (S-4800, Hitachi, Tokoyo, Japan) and transmission electron microscopy (TEM) imaging (JEOL2100 HR, Tokoyo, Japan). Surface element distribution was observed via SEM-EDS. with surface element distribution observed via SEM-EDS. Samples were analyzed using a Bruker D8 Advance X-ray diffraction (XRD) (D8 Advance, Berlin, Germany) with a Cu Kα source at 40 kV and 40 mA. Diffraction peak intensities were recorded within the 10–80° (2θ) range at 0.02° increments. Fourier transform infrared spectroscopy (FTIR) (Bruker Vertex 70, Berlin, Germany) analyzed the infrared spectra of the material across the 400–4000 cm^−1^ wavelength range. X-ray photoelectron spectroscopy (XPS) analysis (AXIS SUPRA, Shimadzu Kratos, Kyoto, Japan) was performed to determine the valence states of surface elements.

### 2.3. Performance Evaluation of MEL-Cu-6HNA for Reactive Red 3BS

To evaluate the degradation performance of the composite MEL-Cu-6HNA catalyst in the BAP system, degradation experiments were conducted in aqueous solutions using Active Red 3BS as a model pollutant. All degradation experiments were conducted in 50 mL colorimetric tubes. Reaction conditions (catalyst concentration, dye concentration, hydrogen peroxide and sodium bicarbonate concentrations, temperature) were systematically optimized based on previously reported experimental protocols for bicarbonate-activated hydrogen peroxide systems [17,18,19]. The absorbance of the solution at 540 nm was measured using a UV-visible spectrophotometer, and the dye concentration in the solution was calculated via a standard curve. The decolorization rate (%) was calculated using the following formula:

(1) $$Q=\frac{C_{0}-C_{1}}{C_{0}}\times 100\%=\frac{A_{0}-A_{1}}{A_{0}}\times 100\%$$

where *Q* is the decolorization rate (%), *C*_1_ is the dye concentration after reaction (mg/L), *C*_0_ is the dye concentration before reaction (mg/L), *A*_1_ is the absorbance of the solution after reaction, and *A*_0_ is the absorbance of the dye before reaction.

After selecting the optimal conditions identified in previous studies, EPR (Electron Paramagnetic Resonance) analysis was conducted using TEMP and DMPO as representative scavengers for SO_4_^2−^, ^1^O_2_, ·O_2_, and ·OH, respectively, to better understand the degradation mechanism of MEL-Cu-6HNA on Reactive Red 3BS.

## 3. Research Findings

### 3.1. Characterization of MEL-Cu-6HNA

**Figure 2:**
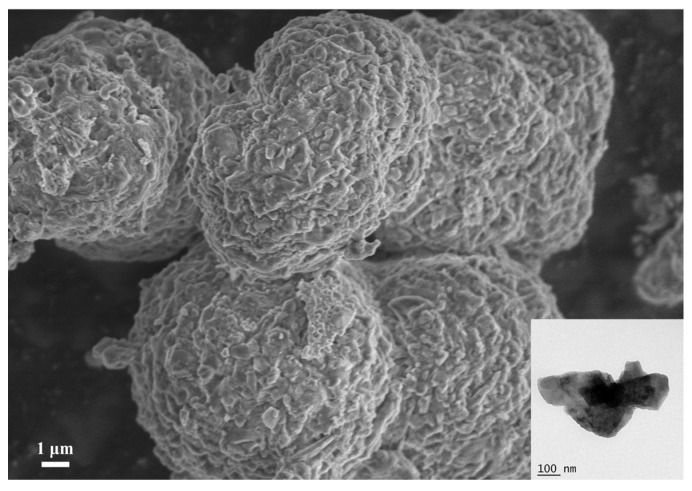
SEM image of MEL-Cu-6HNA (scale bar 1 µm). Inset: TEM image of MEL-Cu-6HNA (scale bar 100 nm).

The MEL-Cu-6HNA composite was characterized using scanning electron microscopy (SEM), with results shown in Figure 2 The MEL-Cu-6HNA precursor underwent hydrothermal self-assembly to form a uniform porous spherical structure. The sample exhibited a regular spherical morphology overall, with the surface of the spheres covered by distinct wrinkles and pores, forming a typical porous agglomerate structure. This highly ordered morphology originates from the synergistic coordination of Cu^2+^ ions with the amino groups of melamine (MEL) and the carboxyl/hydroxyl groups of 6HNA. This coordination forms a rigid supramolecular framework through hydrogen bonding and π-π stacking interactions [20,21], ultimately resulting in short-range ordered porous spheres. Transmission electron microscopy (TEM, TEM map in the inset of Figure 2) clearly reveals that the material consists of primary nanoparticles ranging in size from 20 to 70 nanometers, meeting the internationally recognized definition of nanomaterials, thereby confirming its nanoscale properties.

**Figure 3:**
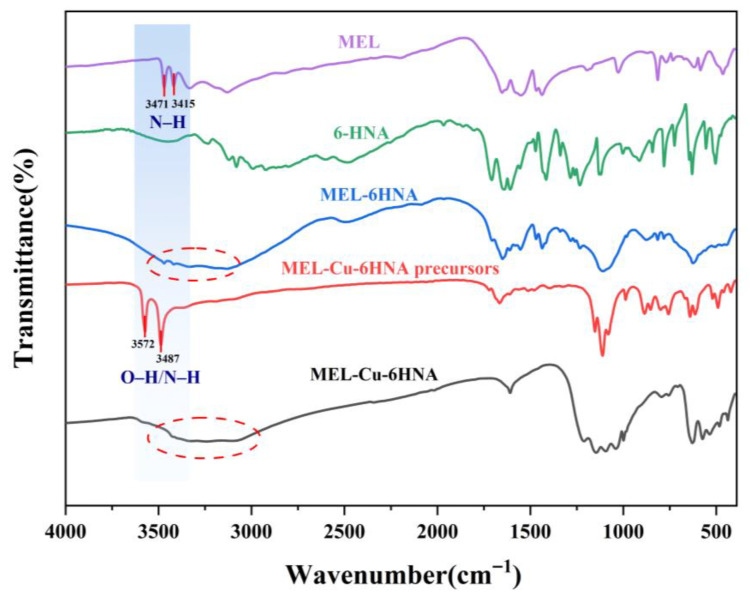
FTIR spectra of melamine (MEL), 6-hydroxynicotinic acid (6-HNA), MEL-6HNA Self-Assembly, MEL-Cu-6HNA precursors, and the final MEL-Cu-6HNA composite.

Figure 3 presents the FT-IR spectra of MEL, 6-HNA, Mel-6HNA Self-Assembly, the Mel-Cu-6HNA precursor, and the final Mel-Cu-6HNA material. During the preparation process, Cu^2+^ preferentially coordinates with the hydroxyl and carboxyl groups of 6-HNA as well as the amino group of MEL, thereby disrupting the original hydrogen bonding network present in the pure ligands [22]. This coordination leads to significant broadening of the O–H and N–H stretching vibration peaks in the range of 3200–3600 cm^−1^, along with the emergence of high-frequency shoulder peaks (e.g., N–H at 3471 and 3415 cm^−1^, and O–H⋯H at 3571 and 3487 cm^−1^), which indicate the formation of an extensive hydrogen bonding network involving N–H⋯O and O–H⋯N interactions. These interactions facilitate the construction of supramolecular architectures under hydrothermal conditions. Subsequently, high-temperature calcination in air at 500 °C induces deep carbonization, deamination, and dehydroxylation of the organic components [23]. The characteristic absorption bands of the Mel-Cu-6HNA structure are largely absent in this region, with only a weak vibration peak around 1620 cm^−1^—attributable to C=N and C=O functional groups—retained in the fingerprint region, suggesting that most –NH_2_ and –OH groups have been effectively removed. As a result, the formation of Cu species embedded within an N-doped carbon matrix is accomplished. Residual nitrogen- and oxygen-containing functional groups may serve as additional active sites, potentially enhancing the material’s catalytic or adsorption performance. The evolution observed in the FT-IR spectra clearly demonstrates how the sequential strategy—from hydrothermal preassembly to high-temperature calcination—enables precise regulation of hydrogen bond network formation and decomposition, thus facilitating structural optimization and performance enhancement of the target material.

**Figure 4:**
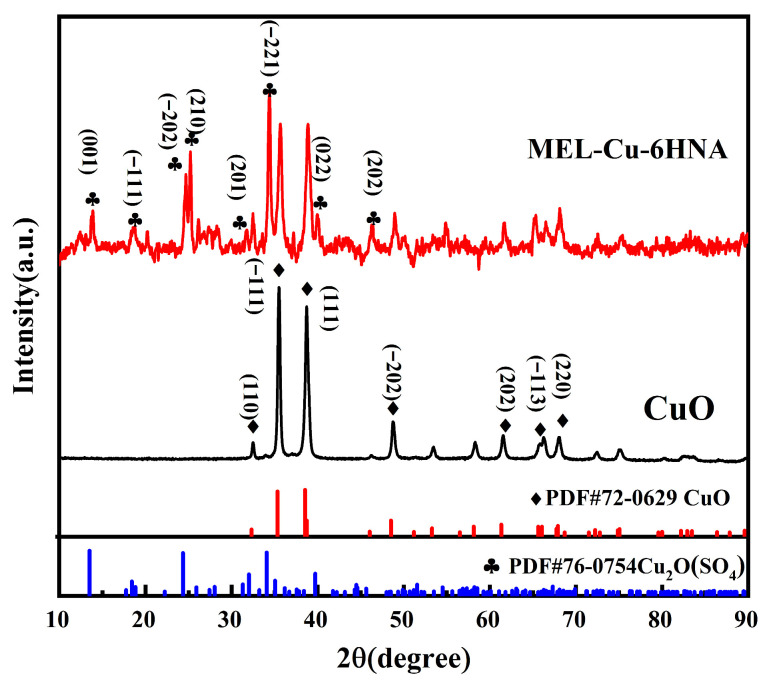
X-ray diffraction (XRD) patterns of MEL-Cu-6HNA composite material and CuO.

To determine the crystal structure of the prepared material, we conducted X-ray diffraction (XRD) analysis. As shown in Figure 4, the shape of MEL-Cu-HNA peak is sharp and well-defined outlines, indicating that the composite material possesses high crystallinity. Peaks appeared at 2θ = 13.84°, 18.64°, 24.665°, 25.19°, 32.441°, 34.426°, 35.613°, 38.805°, 39.89°, 46.397°, 49.016°, 61.744°, 65.141°, and 68.7°, corresponding to (001), (−111), (−202), (210), (201), (−221), (−111), (111), (022), (202), (−202), (202), (−113), and (220) crystal planes. Comparison with standard patterns (PDF#: 76-0754) identifies strong diffraction peaks at 2θ ≈ 14.8°, 25.0°, and 29.2° as characteristic of Cu_2_O(SO_4_) [24]. At elevated temperatures, SO_4_^2−^ groups resist complete decomposition, acting as templates to stabilize partially reduced Cu^+^ ions and form this composite sulfate phase. To further determine the phase composition, we prepared pure CuO as a control. Its XRD pattern (Figure S1) matches the standard monoclinic CuO (PDF#72-0629) with strong peaks at 35.5° (−111) and 38.7° (111). In contrast, these peaks appear only in the very weak shoulder of MEL-Cu-6HNA, indicating that CuO is a minor secondary phase of partial surface oxidation. These dominant peaks are consistent with those reported for copper (I) sulfate (PDF#76-0754), and XPS evidence further supports the predominant Cu^+^ and S^+^ in copper sulfate species. This Cu^+^ rich phase, stabilized by a sulfate template strategy and gentle calcination, is essential for efficient REDOX cycling of Cu^+^/Cu^2+^ during catalysis. XRD results confirm that pyrolysis of the MEL-Cu-6HNA precursor at 500 °C in air successfully produced a composite material with Cu_2_O(SO_4_) as the primary phase and CuO as a secondary crystalline phase. This phase combination is the inevitable result of the synergistic interaction between the specific pyrolysis atmosphere, temperature, and sulfate ions in the precursor.

**Figure 5:**
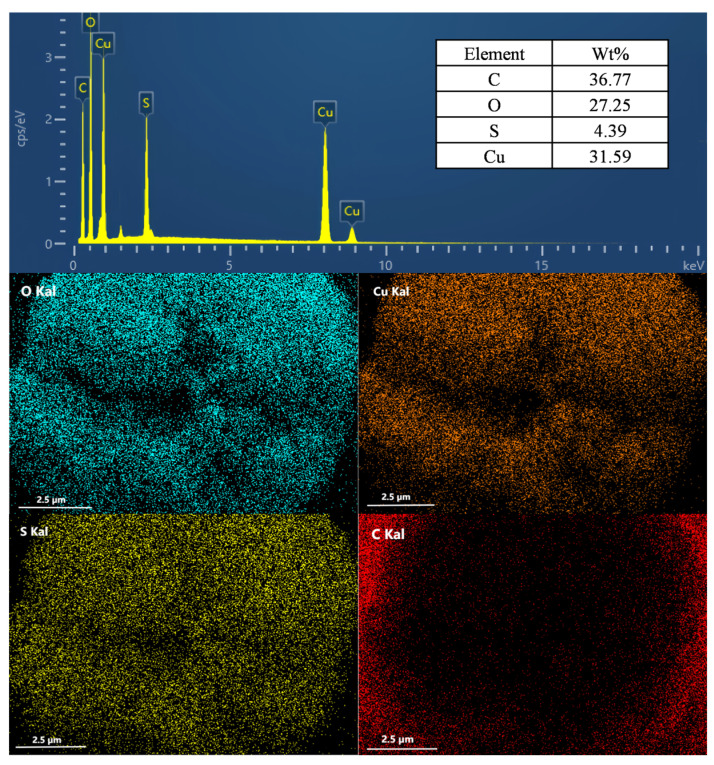
EDS-mapping in MEL-Cu-6HNA.

To confirm the distribution and composition of elements, we performed elemental mapping analysis on the material using energy-dispersive X-ray spectroscopy (EDS), with results shown in Figure 5. The EDS spectrum indicates that the material primarily contains C, O, S, and Cu elements, with mass fractions of 36.77%, 27.25%, 4.39%, and 31.59%, respectively. The elements are uniformly distributed within the spherical structure, with no significant elemental segregation or separation observed. This result strongly corroborates the XRD analysis. XRD has confirmed that the main phase of the material is Cu_2_O(SO_4_), containing a small amount of CuO. The S element detected by EDS corresponds precisely to the SO_4_^2−^ component within the Cu_2_O(SO_4_) phase revealed by XRD; while the Cu element originates from both Cu_2_O(SO_4_) and CuO crystalline phases. The uniform distribution of Cu and S elements within the carbon framework, as shown by EDS mapping, visually demonstrates that the active Cu_2_O(SO_4_) phase is not segregated into large particles but is highly dispersed throughout the N-doped carbon substrate [25,26,27]. This uniformly distributed microstructure directly reflects the synergistic interaction between the in situ growth mechanism during hydrothermal processing and subsequent pyrolysis. This mechanism ensures that copper species and sulfate ions originating from the precursor are in situ-fixed and dispersed during carbonization, forming a composite structure dominated by Cu_2_O(SO_4_) with CuO as a secondary crystalline phase, rather than a simple physical mixture. Consequently, the MEL-Cu-6HNA composite successfully constructs a porous spherical structure with uniform loading of active components. This synergistic effect between phase composition and microstructure provides a robust structural foundation for the material to exhibit outstanding performance in subsequent applications.

**Figure 6:**
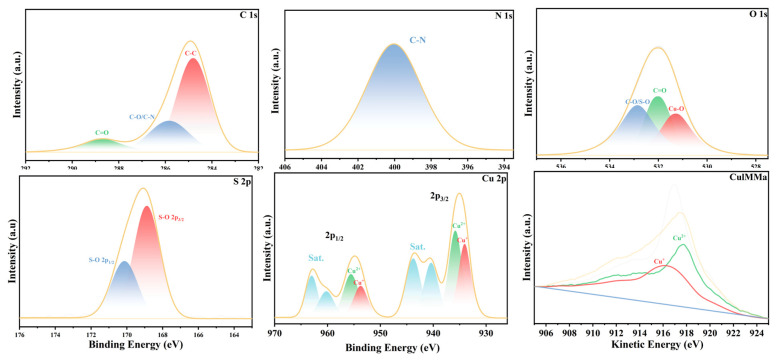
X-ray photoelectron spectroscopy (XPS) spectrum of the MEL-Cu-6HNA composite material.

Figure 6 shows the X-ray photoelectron spectrum (XPS) of MEL-Cu-6HNA. To further investigate the chemical states of surface elements, we performed XPS analysis on the MEL-Cu-6HNA sample. The results in Figure 5 clearly indicate the presence of C, N, O, Cu, and S elements on the material surface, consistent with the elemental composition analysis from EDS. A high-resolution spectrum of the Cu 2p line is presented. A main peak is observed at a binding energy of 932.4 eV, with no significant satellite peaks appearing near 942.0 eV. This indicates that copper in the sample primarily exists in the Cu^+^ valence state, corresponding to the copper species in Cu_2_O(SO_4_). Additionally, a faint shoulder peak with a binding energy near 933.6 eV is present, attributable to Cu^2+^. This corresponds to the minor CuO sub-crystalline phase detected by XRD. Peak fitting estimates the relative content of Cu^+^ at approximately 78%, further confirming Cu_2_O(SO_4_) as the dominant phase on the material surface. The S 2p spectrum exhibits a sharp characteristic peak at a binding energy of 168.5 eV, a position typical for the S^6+^ signal in SO_4_^2−^ [28]. This result provides direct valence evidence for the SO_4_^2−^ vibrational peak at 1100 cm^−1^ observed in FTIR, confirming that sulfate ions have been successfully incorporated into the material structure rather than merely physically adsorbed. The peak at 400.2 eV in the N 1s spectrum corresponds to pyrrolic nitrogen [29], originating from melamine and 6-HNA ligands. These were incorporated into the carbon framework during pyrolysis, not only enhancing the material’s conductivity but also potentially serving as catalytic active sites that synergistically improve the material’s overall performance.

This study successfully synthesized nitrogen-doped carbon-supported copper(II) sulfate oxo-complex materials using MEL-Cu-6HNA as a precursor through a simple two-step “hydrothermal self-assembly–pyrolysis” process. XRD analysis confirmed that under 500 °C air pyrolysis conditions, the material formed a unique phase composition dominated by Cu_2_O(SO_4_) with CuO as a secondary phase—a result of synergistic effects between the sulfate template and air atmosphere. FTIR and EDS analyses further verified the successful introduction of SO_4_^2−^ and the uniform dispersion of all elements within the carbon framework, respectively, from the perspectives of functional group vibrations and elemental distribution. Crucially, XPS analysis precisely verified the coexistence of Cu^+^ (corresponding to Cu_2_O(SO_4_)) and Cu^2+^ (corresponding to CuO) at the valence level, while also revealing the presence of nitrogen-containing functional groups. Collectively, these results demonstrate that the process not only achieves effective composite formation between copper species and nitrogen-doped carbon matrices but also constructs composite materials with abundant active sites and uniform microstructures through precise phase control. The unique phase composition and surface chemical structure provide a robust material foundation for the material’s outstanding performance in fields such as environmental remediation.

### 3.2. Catalytic Decolorization Performance Analysis

In recent years, advanced oxidation processes (AOPs) activated by hydrogen peroxide have garnered significant attention for treating dyeing and printing wastewater. Among these, Fenton-like systems demonstrate superior decontamination potential due to their ability to generate highly reactive radicals such as ·OH and SO_4_^2−^ under mild conditions. However, traditional homogeneous Fenton systems face limitations including narrow pH ranges, metal ion leaching, and secondary sludge pollution, making them unsuitable for practical applications. Therefore, developing highly efficient, stable, and recyclable heterogeneous catalysts has become a current research focus [30]. This study constructed a MEL-Cu-6HNA composite structure, which demonstrated excellent decolorization performance in the HCO_3_^−^/H_2_O_2_ system, confirming the application prospects of copper-based heterogeneous catalysts in textile wastewater treatment.

**Figure 7:**
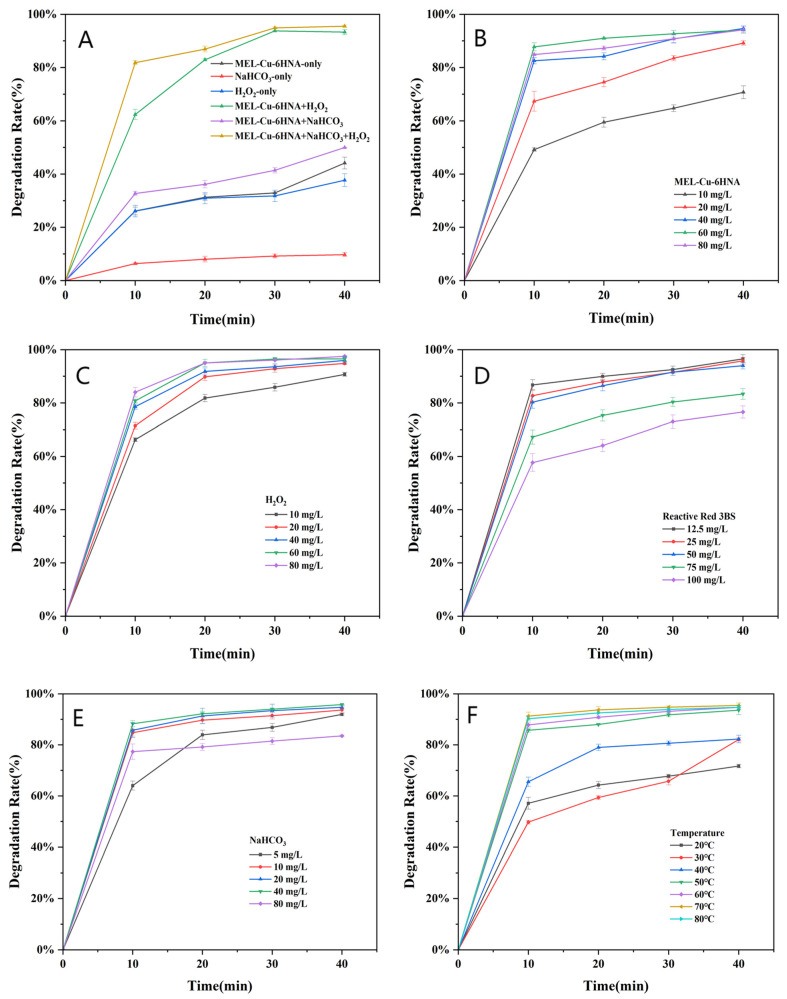
(**A**) Decolorization rates of Reactive Red 3BS under different catalytic systems; (**B**) Decolorization rates of Reactive Red 3BS as a function of the concentration of MEL-Cu-6HNA; (**C**) Decolorization rates of Reactive Red 3BS as a function of the dye concentration; (**D**) Decolorization rates of Reactive Red 3BS as a function of the H_2_O_2_ concentration; (**E**) Decolorization rates of Reactive Red 3BS as a function of the NaHCO_3_ concentration; (**F**) Decolorization rates of Reactive Red 3BS as a function of the reaction temperature.

As shown in Figure 7A, the decolorization rate was less than 10% when NaHCO_3_ was used alone, and less than 60% when either MEL-Cu-6HNA or H_2_O_2_ was used alone, indicating that a single component cannot effectively drive the degradation of Reactive Red 3BS. When MEL-Cu-6HNA synergized with H_2_O_2_, decolorization exceeded 80% within 30 min, demonstrating catalytic activity. With all three components present simultaneously, decolorization rapidly surpassed 85% within 10 min and approached complete removal after 30 min—significantly outperforming other control systems. This indicates that MEL-Cu-6HNA plays a pivotal role in this BAP system, significantly enhancing dye degradation efficiency by promoting sustained radical generation. Compared to previously reported single-phase catalysts like CuO and Cu_2_O, the CuO/Cu_2_O(SO_4_) composite structure developed in this study demonstrates superiority in both reaction rate and final efficiency. This indicates that the biphasic structure exhibits synergistic effects in enhancing electron transfer and the generation of active species.

During further condition optimization, a variable control method determined optimal parameters: 50 mg/L Reactive Red 3BS, 10 mg/L NaHCO_3_, 40 mg/L H_2_O_2_, 50 °C temperature, with MEL-Cu-6HNA concentration as the variable (10–80 mg/L). Figure 7B shows decolorization efficiency follows a “first increase, then plateau” trend. At low doses (10 mg/L), the reaction rate was limited. When the catalyst concentration increased to 40 mg/L, the decolorization rate exceeded 80% within 10 min and surpassed 90% within 30 min, demonstrating optimal performance. Further increases to 60–80 mg/L did not significantly improve the final efficiency, indicating that oxidant supply became the limiting factor. This aligns with Chai et al.’s findings on the high activation efficiency of copper-based catalysts in BAP systems [31], demonstrating that efficient degradation can be achieved with minimal catalyst usage, offering economic advantages.

Variations in initial dye concentration also significantly impacted system performance (Figure 7C). Using a control variable method, optimal conditions were determined as: NaHCO_3_ at 10 mg/L, H_2_O_2_ at 40 mg/L, temperature at 50 °C, and MEL-Cu-6HNA concentration at 40 mg/L. When the Reactive Red 3BS concentration increased from 12.5 mg/L to 100 mg/L, the decolorization rate decreased from 95% to 72%. This indicates that elevated pollutant concentrations lead to a relative shortage of free radicals, thereby reducing reaction efficiency. This trend aligns with findings from Wang et al.’s study on AO7 dye [32], suggesting this system is more suitable for treating low-to-medium concentration textile wastewater.

Regarding oxidant dosage, H_2_O_2_ (40 mg/L) and NaHCO_3_ (10 mg/L) exhibited optimal concentrations. Insufficient H_2_O_2_ limited radical generation, markedly reducing reaction rates; while excessively high concentrations reduced utilization due to ineffective decomposition or radical quenching. Similarly, increasing NaHCO_3_ concentration to moderate levels significantly accelerated degradation rates. However, concentrations exceeding 60 mg/L caused turbidity and precipitation in the system, likely due to the formation of byproducts such as Cu(OH)_2_ from the interaction between NaHCO_3_ and Cu^2+^, which impaired the utilization efficiency of active sites. This aligns with Li et al.’s [33] conclusion regarding the saturation threshold of H_2_O_2_ concentration, indicating that rational regulation of oxidant ratios is crucial for achieving efficient degradation.

Temperature represents another critical factor (Figure 7F). Experimental results demonstrate that the reaction rate increases with rising temperature, reaching an optimum at 50 °C with a decolorization rate approaching 95% within 30 min. However, efficiency declines when temperature is further elevated to 70–80 °C, likely due to accelerated decomposition of H_2_O_2_ at high temperatures, resulting in reduced generation of effective radicals. This result reveals the endothermic nature of the BAP system, indicating that excessively high temperatures are detrimental to maintaining system stability. 50 °C represents the optimal choice balancing reaction rate and energy efficiency.

A shown in Table 1, most reported copper-based catalysts rely on H_2_O_2_ or PMS as oxidants, requiring higher catalyst doses (0.1–0.6 g/L or equivalent) and longer reaction times (30–75 min) to achieve comparable decolorization rates. In contrast, the MEL-Cu-6HNA catalyst exhibits outstanding activity under low doses (40 mg/L) of NaHCO_3_/H_2_O_2_. This study achieved 95% decolorization of Reactive Res 3BS (50 mg/L) within just 30 min. MEL-Cu-6HNA exhibits significantly superior decolorization performance compared to single-component or dual-component systems in the HCO_3_^−^/H_2_O_2_ system. Its high efficiency stems not only from enhanced radical generation rates under optimized conditions but also from the synergistic effects of the CuO/Cu_2_O(SO_4_) composite structure: on one hand, the Cu^2+^/Cu^+^ cycle accelerates H_2_O_2_ activation, promoting sustained generation of ROS such as ·OH and SO_4_·^−^; while the three-dimensional porous framework with uniformly distributed active sites facilitates reactant diffusion and electron transfer, lowering energy barriers and suppressing side reactions. Compared to existing reports, this system achieves rapid decolorization exceeding 90% under mild conditions, fully demonstrating its potential for treating dyeing wastewater.

### 3.3. Mineralization Capacity of MEL-Cu-6HNA Toward Reactive Red 3BS in the BAP System

**Figure 8:**
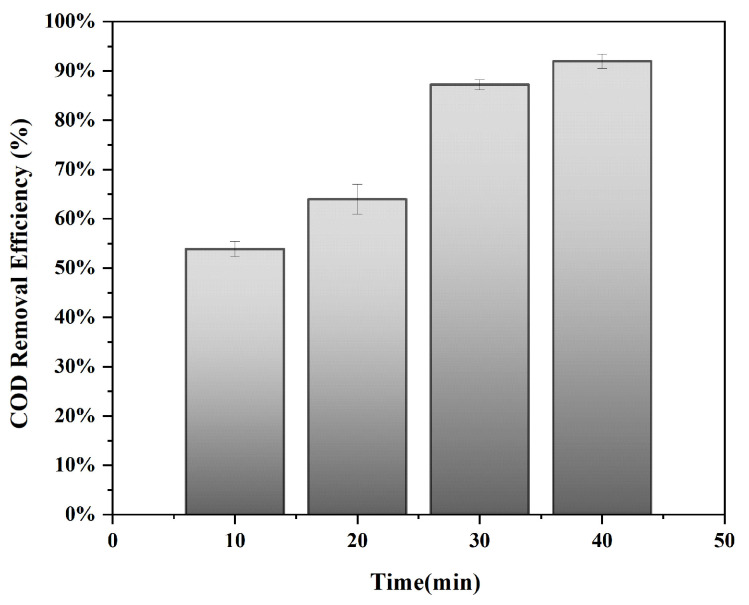
COD removal efficiency of Reactive Red 3BS by MEL-Cu-6HNA in the BAP system.

To further evaluate the mineralization capacity of MEL-Cu-6HNA in the BAP system, the changes in chemical oxygen demand (COD) of the Reactive Red 3BS solution were measured at different reaction times, with results shown in Figure 8. It can be observed that the COD value gradually decreased as the reaction progressed. The COD removal rate reached approximately 85% at 30 min, which was comparable to the simultaneously measured decolorization rate (>90%) but slightly lower than it. This indicates that while the system achieved rapid decolorization, it was also accompanied by the deep degradation of most organic pollutants, with only a small amount of intermediate products remaining unmineralized.

### 3.4. Analysis of Decolorizing Species Activity

**Figure 9:**
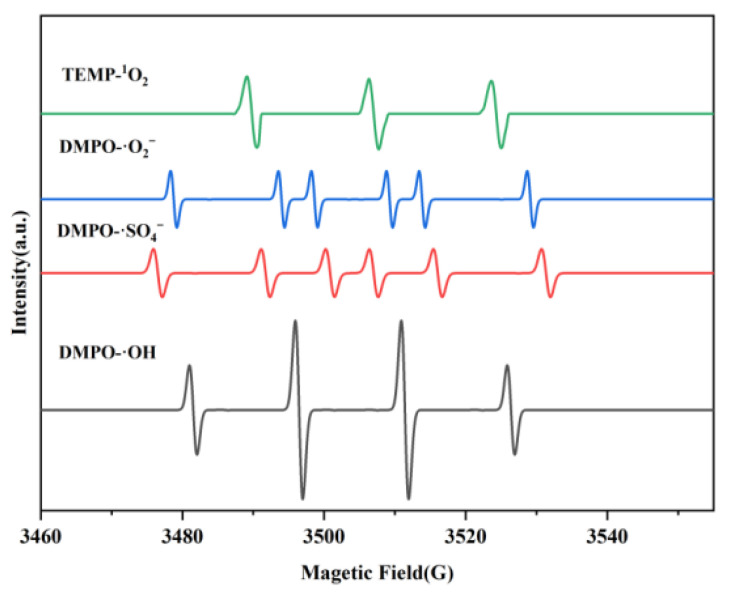
EPR spectrum of the degradation process of Reactive Red 3BS by MEL-Cu-6HNA in the BAP system.

EPR spectra obtained using 2,2,6,6-tetramethylpiperidine (TEMP) and 5,5-dimethyl-1-pyrrolidine-N-oxide (DMPO) as radical scavengers for ^1^O_2_, ·OH, SO_4_·^−^, and ·O_2_^−^, respectively, are shown in Figure 9. It can be observed that DMPO-·OH exhibits a characteristic peak, confirming the presence of ·OH; DMPO-SO_4_·^−^ displays a hyperfine-split six-line pattern, indicating the presence of SO_4_·^−^ [39], possibly originating from secondary reactions of sulfate ions in Cu_2_O(SO_4_); DMPO-·O_2_^−^ shows a weaker four-line signal, suggesting a minor contribution from ·O_2_^−^; TEMP-1O_2_ exhibits a 1:1:1 triplet signal resulting from TEMP binding with ^1^O_2_ [40]. The signal intensity order DMPO-·OH > DMPO-SO_4_·^−^ > TEMP-^1^O_2_ > DMPO-·O_2_^−^ emphasizes the dominant role of ·OH in the oxidation process. Based on EPR identification and copper oxidation state synergistic effects, the following reaction mechanism is proposed. The CuO/Cu_2_O(SO_4_) structure in MEL-Cu-6HNA activates H_2_O_2_ through Cu(II)/Cu(I) cycling, generating reactive oxygen species (ROS) that drive dye oxidative degradation.

·OH + SO_4_^2−^ → SO_4_·^−^ + OH^−^

Cu(II) + H_2_O_2_ → Cu(I) + HO_2_· + H^+^

Cu(I) + H_2_O_2_ → Cu(II) + ·OH + OH^−^

EPR results confirm ·OH as the primary ROS, with SO_4_·, ^1^O_2_, and ·O_2_^−^ playing auxiliary roles. Studies indicate that the reaction rate between OH^−^ and HCO_3_^−^ to form CO_3_·^−^ is significantly lower than the rate of direct ·OH generation from OH^−^ and H_2_O_2_. Consequently, most ·OH is “consumed” by HCO_3_^−^, resulting in extremely low CO_3_·^−^ concentrations that are difficult to detect via EPR [41]. The three-dimensional structure of CuO/Cu_2_O(SO_4_) enhances the dispersion and stability of dual Cu sites, promoting the aforementioned cycle. As an azo dye, the decolorization of Reactive Red 3BS relies on ROS attacking its molecular structure. The high oxidizing power of ·OH preferentially breaks azo bonds (-N=N-) and aromatic rings, destroying the chromophore; SO_4_^2−^ selectively oxidizes electron-rich sites, enhancing degradation efficiency; ^1^O_2_ and ·O_2_^−^ assist in oxidizing unsaturated bonds, promoting complete molecular cleavage. The 95% decolorization rate achieved experimentally stems from this multi-ROS synergistic mechanism, which elevates the catalytic performance of the BAP system. This mechanism confirms that MEL-Cu-6HNA efficiently generates ROS through copper oxidation-reduction cycles, driving the oxidative decolorization of Reactive Red 3BS, thereby providing a theoretical foundation for dyeing wastewater treatment.

## 4. Conclusions

This study successfully constructed the MEL-Cu-6HNA material with a CuO/Cu_2_O(SO_4_) composite structure based on the supramolecular self-assembly strategy of melamine and 6-hydroxy-nicotinic acid. Systematic characterization revealed that this material possesses a three-dimensional porous framework and uniformly distributed active sites, forming a stable composite structure with biphasic synergy. Leveraging Cu^2+^/Cu^+^ cycling and interfacial electron transfer effects, MEL-Cu-6HNA exhibits outstanding catalytic performance in the BAP system: under optimized conditions (catalyst concentration 40 mg/L, NaHCO_3_ 10 mg/L, H_2_O_2_ 40 mg/L, 50 °C), the decolorization rate of Reactive Red 3BS exceeded 90% within 30 min, with COD removal reaching 85%, significantly outperforming traditional single-component systems. Mechanistic studies indicate that ·OH is the dominant active species, while SO_4_·^−^, ^1^O_2_, and ·O_2_^−^ play auxiliary roles. The synergistic action of multiple ROS rapidly disrupts azo bonds and aromatic structures, promoting deep oxidation and mineralization of organic pollutants. The results demonstrate that the CuO/Cu_2_O(SO_4_) composite structure not only resolves the issues of insufficient activity and poor stability in single-phase copper-based catalysts but also enables efficient operation of Fenton-like systems under neutral to weakly acidic conditions. Additionally, the long-term stability of MEL-Cu-6HNA from the same batch was preliminarily evaluated by storing the material at ambient conditions for several months at room temperature (Table S1). Monthly degradation experiments conducted under optimized conditions (40 mg/L catalyst, 50 mg/L Active Red 3BS, 40 mg/L H_2_O_2_, 10 mg/L NaHCO_3_, 50 °C, 30 min) consistently maintained decolorization efficiencies between 94 and 97%, highlighting the composite’s robust durability and practical application potential for dye wastewater treatment. In summary, the MEL-Cu-6HNA material proposed in this study demonstrates innovation in both structural design and catalytic performance. It not only offers new insights for the controlled synthesis and functional optimization of copper-based composites but also provides theoretical foundations and practical references for the green and efficient treatment of textile dyeing wastewater.

## Figures and Tables

**Table 1:** Comparison of Dye Degradation Performance with Typical Copper-Based.

Catalytic	Catalyst Concentration	Oxidizing Agent	Pollutants	Time (Min)	Decolorization Rate (%)	References
CuO NP	0.6 mM	H_2_O_2_	Methylene blue, 53.5 μM	75	99.6%	[34]
Cu2O–Cu/C	0.1 M	H_2_O_2_	Methyl Orange, 50 mg/L	60	100%	[35]
CuCo-CTs	0.1 g/L	Peroxymonosulfate	Reactive Violet 5, -	30	90%	[36]
Cu/FeNPs@PC	0.2 g/L	Peroxymonosulfate	Rhodamine B, -	18	98.4%	[37]
Cu-Co/C	0.5 g/L	Peroxymonosulfate	Orange II, 50 mg/L	30	90.4%	[38]
MEL-Cu-6HNA	40 mg/L	NaHCO_3_/H_2_O_2_	Reactive Res 3BS, 50 mg/L	30	95%	This work

## Data Availability

The original contributions presented in this study are included in the article/Supplementary Material. Further inquiries can be directed to the corresponding author.

## Supplementary Materials

The following supporting information can be downloaded at: https://www.mdpi.com/article/10.3390/nano16020111/s1, Figure S1: XRD pattern of CuO; Table S1. Long-term storage stability of MEL-Cu-6HNA.
