# Supplementary material for: Study on the Efficiency and Mechanism of a Novel Copper-Based Composite Material Activated by Supramolecular Self-Assembly for Degrading Reactive Red 3BS
Source: Nanomaterials (Basel). 2026 Jan 15;16(2):111. doi: 10.3390/nano16020111 (PMC12843984; doi:10.3390/nano16020111)
Supplement: Supplementary file 1 [file nanomaterials-16-00111-s001.zip › nanomaterials-4040111-supplementary.pdf]

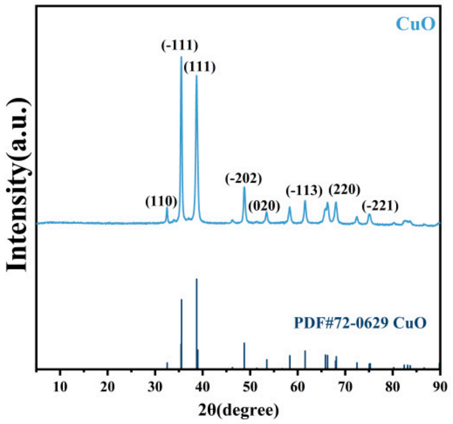

Figure S1. XRD pattern of CuO

Figure S1. XRD pattern of pure CuO control prepared by calcining compared with standard PDF#72-0629.

Table S1. Long-term storage stability of MEL-Cu-6HNA

Material Preparation Time	Material Experiment Time	MEL-Cu-6HNA Concentration （mg/L）	$NaHCO_3$ Concentration （mg/L）	H_2O_2 Concentration mg/L	Tempe rature ℃	Decolorization Rate %
23/04/2025	25/04/2025	40	10	40	50	94.55%
23/04/2025	28/04/2025	40	10	40	50	95.34%
23/04/2025	21/05/2025	40	10	40	50	95.38%
23/04/2025	28/05/2025	40	10	40	50	94.96%
23/04/2025	14/06/2025	40	10	40	50	94.10%
23/04/2025	27/06/2025	40	10	40	50	95.02%
23/04/2025	10/07/2025	40	10	40	50	95.82%
23/04/2025	29/07/2025	40	10	40	50	96.06%
23/04/2025	18/08/2025	40	10	40	50	96.88%
23/04/2025	26/08/2025	40	10	40	50	94.77%
23/04/2025	26/09/2025	40	10	40	50	95.78%
23/04/2025	30/09/2025	40	10	40	50	95.34%
